# Supplementary material for: Financial markets value skillful forecasts of seasonal climate
Source: Nat Commun. 2024 May 14;15:4059. doi: 10.1038/s41467-024-48420-z (PMC11093977; doi:10.1038/s41467-024-48420-z)
Supplement: Supplementary file 1 — Supplementary Information [file 41467_2024_48420_MOESM1_ESM.pdf]

Supplementary Information for "Financial Markets Value Skillful Forecasts of Seasonal Climate"

Derek Lemoine[1,2] and Sarah Kapnick[3,4]

2024

[1] University of Arizona, 1130 E. Helen St, McClelland 401, Tucson, AZ 85721, USA

[2] National Bureau of Economic Research, 1050 Massachusetts Ave, Unit 32, Cambridge, MA 02138, USA

[3] National Oceanic and Atmospheric Administration, 1401 Constitution Avenue NW, Room 5128, Washington, DC 20230, USA

[4] Formerly Geophysical Fluid Dynamics Laboratory, National Oceanic and Atmospheric Administration, Princeton, New Jersey 08540, USA

Supplementary Discussion 1 proves that the variance of a future stock price must decline on average following the release of a seasonal outlook. Supplementary Discussion 2 describes the seasonal outlooks we study. Supplementary Discussion 3 contains additional details on sample construction. Supplementary Discussion 4 discusses identification. Supplementary Discussion 5 reports additional results. Supplementary Discussion 6 assesses sensitivity to alternate targets for measuring ENSO forecast skill.

Supplementary Discussion 1: Proof that Uncertainty Must Decline on Average Across Releases of a Relevant Outlook

Let T be the date an option expires and let $\tau < T$ be the date an outlook is released. The time t stock price is S_t. Let W_τ denote the contents of the outlook, which are known as of τ but not any earlier. From the law of total variance,

$$Var[S_T] = E[Var[S_T|W_\tau]] + Var[E[S_T|W_\tau]].$$

Rearranging,

$$E[Var[S_T|W_\tau]] - Var[S_T] = -Var[E[S_T|W_\tau]].$$

The first term on the left-hand side is traders' variance of S_T averaged over all the possible outlook contents W_τ, and the second term on the left-hand side is traders' variance of S_T without knowing the outlook contents W_τ. Either variance includes randomness in weather that might occur by time T, all other random shocks that might affect firm value by time T, and the possibility that outlooks' predictions are revised by information arriving before time T. However, only the second variance on the left-hand side includes uncertainty about what the outlook will say.

Using iterated expectations, this equation implies

$$E\big[Var[S_T|W_\tau] - Var[S_T]\big] = -Var[E[S_T|W_\tau]].$$

The left-hand side is the expected change in traders' variance upon the release of an outlook. If we observe many outlook releases, the left-hand side gives the average change in traders' variance upon the release of the outlook. The right-hand side is the negative of the variance in the stock price induced by the possible outlooks. The right-hand side is weakly negative, and it is strictly negative if the outlook's estimates might be relevant for the stock price. Therefore traders' perceived variance of S_T should fall on average upon the release of an outlook if the outlook is potentially informative for stock prices, and traders' perceived variance of S_T should not change on average otherwise. We test for the average decline in variance by using the change in the variance implied by options' prices.

For completeness, one might wonder how our formalism would apply to a seasonal outlook that traders perceived to have no skill. The release of such an outlook would not move stock prices at all, so that $E[S_T|W_\tau]$ is independent of W_τ. In that case, $Var[E[S_T|W_\tau]] = 0$. So the average change in traders' variance upon the release of the outlook must also be 0: we should not find any effect of releasing an outlook. This possibility of no skill is included within the null hypothesis we test.

Supplementary Discussion 2: Outlooks of Interest

We consider three main seasonal products developed by the National Oceanic and Atmospheric Administration (NOAA): Atlantic Hurricane Seasonal Outlook, U.S. Winter Outlook, and El Niño Southern Oscillation (ENSO) Forecast. Each is released according to a regular, announced schedule with strict information management. Non-disclosure agreements (NDAs) were broadly implemented for the hurricane outlook beginning in 2012. The ENSO and winter outlooks do not use NDAs, but participants are covered under NOAA ethics rules and receive a reminder the day before a release not to disclose its contents until they are made public. Each release is a cross-platform media event using various NOAA websites (NOAA.gov, Cli-

mate.gov, cpc.ncep.noaa.gov) and, since 2008, social media platforms. These releases make national headlines.

These three products are all "seasonal", providing multi-month predictions.[1] In atmospheric sciences, the word "forecast" is usually used to denote weather-scale time horizons whereas "predictions" are used to denote climate-scale horizons. At the seasonal timescale, there is some mixing of terminology. Throughout this study, we refer to these products as "outlooks" and to the contents of an outlook as its "forecast".

The outlooks have been enhanced over time, with an especially notable change around 2010. Before 2010, the NOAA outlooks were based on a combination (whole or part) of observations, ENSO prediction models, statistical models, and available climate models. In 2011, NOAA put the Climate Forecast System (CFS), a new global prediction model, into production to provide real-time forecasts for the National Weather Service. At the same time, the North American Multi-Model Ensemble (NMME), a seasonal forecasting system consisting of global coupled models from major North American modeling centers was also made operational, with data and summary forecasts provided by the NOAA Climate Prediction Center (*2*). The NMME average has been shown to improve seasonal prediction skill for ENSO over individual models alone (*3*). Additionally, improvements in earth system monitoring, especially the deployment of Argo floats (autonomous drones in the ocean monitoring temperature and salinity) in the 2000s, have facilitated initialization of the subsurface ocean, improving global coupled model seasonal prediction capabilities over time (*4*). Indeed, subsequent research demonstrated predictive skill enhancements in ENSO and hurricanes (*5–8*).

The Atlantic Hurricane Seasonal Outlook includes outlooks for both the Atlantic and East-

[1]The scientific and prediction application community uses two main definitions of "seasonal" timescales. The Weather Research and Forecasting Innovation Act of 2017 (Public Law No: 115-25) defines seasonal prediction as 3 months to 2 years. This is a longer timescale than other definitions of seasonal prediction. For instance, Sub-Seasonal to Seasonal (S2S) prediction is defined as 2 weeks to 1 year in climate science (*1*).

ern Pacific basins.[2] NOAA releases the outlook in May and updates it in August when the Atlantic historically reaches peak activity, archived at `https://www.cpc.ncep.noaa.gov/products/outlooks/hurricane-archive.shtml`. The outlook reports likely ranges of hurricane activity over the full hurricane season, which runs from June 1 through November 30. We use the primary, May release, which has a horizon of 1–7 months.

The U.S. Winter Outlook is typically released on the 3rd Thursday of October. This product reports the probability that each part of the country will experience abnormal seasonal temperatures or precipitation over the coming December through February. A few releases differ from the standard schedule. In 2007, the outlook was released early for the Winter Fuels Outlook Conference and updated later on October 18. In 2008, the outlook was delayed. There is no written record why, but it was potentially due to the Winter Fuels Outlook Conference. In 2013 the outlook was delayed due to a shutdown of the U.S. federal government. This outlook therefore has a horizon of 2–4 months. Outlook dates and information were pulled from press releases on `cpc.ncep.noaa.gov`, `noaa.gov`, and `climate.gov`.

The El Niño Southern Oscillation (ENSO) seasonal outlook has been released on the second Thursday of every month since May 2015. Prior to 2015, the monthly release occurred on the Thursday lying between the 4th and 10th of the month, except in July when it was at the discretion of the ENSO forecast team (due to the U.S. Independence Day holiday falling on July 4th or the nearest weekday). The archive of the ENSO forecasts is available at `www.cpc.ncep.noaa.gov/products/expert_assessment/ENSO_DD_archive.shtml` since 2001. This forecast reports the current state of ENSO and provides predictions out to 9 months. ENSO affects the temperature and heat content of the tropical Pacific and can influence global and regional climate by dynamically altering ocean-atmosphere interactions (i.e. atmospheric cir-

[2] Recent work shows that options markets capitalize the uncertainty reported in forecasts of particular hurricanes' tracks (*9*). We here explore whether they capitalize uncertainty about which seasonal forecasts will be produced.

culation changing patterns of precipitation, temperature, storminess), a phenomenon known as "teleconnections" (*10*). ENSO is often found to be the signal with the most predictive power for other climate variables such as temperature and precipitation (*11*).

Even though ENSO outlooks are released monthly, not all are equally anticipated. In particular, it is widely known that ENSO outlooks' skill is low in the spring, in part because the peak of ENSO (November–January) is still many months away and in part because important stochastic drivers are realized during the boreal spring (*12, 13*). ENSO outlook skill across constant lead times drops in March and April, increases substantially with the June release and does not improve quickly afterward, a phenomenon known as the "spring barrier" (*13–16*). We have reproduced ENSO skill from Figure 2 of (*17*) in supplementary table 1 for reference. This information is used in Figure 4 of the main text to quantify the marginal value of ENSO skill for an October-December target. We focus on the June release because it should be especially informative. This release is around 5–8 months before the peak (November-January) of the forecasted ENSO event.

We also study two non-NOAA outlooks. The Farmers' Almanac's predictions apparently follow a formula that depends on astronomical factors such as sunspot activity, tidal action, and planetary positions—and not on earth observations or numerical weather models (*18*). Similar forecasts have been shown to lack skill (*19*), but this outlook does garner media attention. Its predictions are released around two months before the NOAA Winter Outlook. The Colorado State University (CSU) hurricane outlook is the pioneering seasonal outlook (*20*). Its April release is around a month and a half before the May NOAA Hurricane Outlook. The CSU outlook garners substantial media attention but is less skillful than its NOAA counterpart (*6*). Because these outlooks are released before their NOAA counterparts, the deck is stacked in favor of finding an effect for them but not for the NOAA outlooks: if two outlooks contained the same information, that information should affect markets only when it is first released.

Supplementary table 2 reports the outlook release dates. We collected dates for NOAA outlooks from their individual archives and press releases mentioned above. We collected dates for CSU outlooks from the release archive: `https://tropical.colostate.edu/archive.html`. We collected dates for Farmers' Almanac outlooks by searching for press releases and news articles.

Supplementary Discussion 3: Sample Restrictions

We obtain our final sample by applying the following restrictions. First, for each outlook-year pair, we drop firms that have an earnings announcement on any day in the three-day event window. Second, within the chosen expiration date, we drop all firm-trading day-strike triplets with either a bid of zero or zero open interest. We also drop any anomalous observations with an option price greater than the stock price or with a negative bid-ask spread. Third, for each outlook-year pair, we drop firms that either have no remaining observations on some day in the event window, have fewer than 15 trading days in the estimation window (i.e., beyond the event window) with observations, or have zero volume every day in the estimation and event windows. Fourth, for each outlook-year pair, we drop firms with a quarterly dividend yield greater than 2% over the estimation window and drop firms whose stock price falls below $5 at any point in either the estimation or event windows (*21*).

Supplementary Discussion 4: Identification

β_{fm0} is identified as long as other news that affects uncertainty about future equity prices (net of the controls) is not systematically correlated with the release of NOAA outlooks. One might be concerned about a few different types of news.

First, investors may anticipate earnings reports that come out around the same time as NOAA outlooks. We take care of this concern in two ways (see Methods). First, when estimat-

ing effects for any given outlook, we drop firm-year pairs that have an earnings report within a day of the outlook release. Second, we dummy out the day a company's earnings are reported as well as the day before and after it. So any company-year pairs with an earnings report within a day of a NOAA outlook do not contribute to the estimated effect, and any company-day pairs within a day of an earnings report also do not contribute to the estimated effect.

Second, one might be concerned about several of the most prominent economic announcements made by the Federal Reserve, the Bureau of Labor Statistics, and others. Supplementary table 3 reports the release of major economic news within three days of a seasonal outlook release. It considers seven different types of news releases, which together capture the major interest rate, GDP, employment, and inflation announcements. Entries with a 0 (in bold) indicate that an announcement came out the same day as the NOAA outlook. We see that this coincidence never happened for the June ENSO Outlook, happened five times for the Winter Outlook in 2000–2019 and three times for the Winter Outlook in our 2010–2019 period of interest (with two times being inflation reports and one time an unemployment report), and happened one time for the Hurricane Outlook (an unemployment report). Our June ENSO estimates will not be affected by any of these reports, and our other estimates may not be either.

Third, one might also be concerned by releases of the Unemployment Insurance Weekly Claims Report, which includes initial jobless claims. In our study period, these releases occur on Thursdays. Supplementary table 2 shows that all three NOAA outlooks were nearly always released on a Thursday. If initial jobless claim news were driving our estimated effects then we would expect to detect similar effects for all of the NOAA outlooks. However, we do not in fact detect a significant effect for the NOAA Hurricane Outlook. In addition, all months' ENSO outlooks were released on Thursdays, not just the June outlook (see Supplementary Discussion 2). But Figure 4a in the main text showed that we detect a significant effect only for the June ENSO Outlook. This evidence again makes it unlikely that anticipation of weekly initial jobless

claims data drives our results.

Supplementary Discussion 5: Additional Results

Supplementary table 4 reports the estimates underlying Figure 2 of the main text, along with the number of underlying observations and p-values for a one-sided test of the null hypothesis of a weakly positive effect. The standard errors are similar across outlooks. The different levels of significance are driven by differences in central estimates.

The NOAA Hurricane Outlook's estimates rely on much less data than do the other outlooks' estimates. The NOAA Hurricane Outlook happens to be released relatively far from the expiration date used in the analysis. For instance, the NOAA Hurricane Outlook and the NOAA June ENSO Outlook use the same expiration dates, but the June ENSO Outlook is released 2–3 weeks later (supplementary table 2). The end of the estimation window for the Hurricane Outlook is just a bit too late for the next-earlier expiration date. As options are more thinly traded farther from expiration dates, the Hurricane Outlook loses more observations due to sample restrictions (and what observations there are may be noisier).

Supplementary figure 1 reports the analogues of Figures 2c–d for the other outlooks. Out of all these sectors, only a single effect is significant at the 10% level: Services (Repair/Personal) for the NOAA Hurricane Outlook. We would expect to see at least one by chance. Supplementary figure 2 reports the analogues of Figures 2e–f for the other outlooks (we collected release dates for the Farmers' Almanac only from 2010). We see very different patterns from the main text, with estimates no longer clearly decreasing over time, with fewer significant estimates, and with the significant estimates not clustering in more recent years. (And supplementary table 3 showed that the significant 2015 release for the NOAA Hurricane Outlook corresponded to the release of an unemployment report.) These two figures suggest that the effects detected for the NOAA June ENSO and Winter Outlooks are in fact real effects.

Supplementary figure 3 reports outlooks' average effects on equity markets in the decade (2000–2009) before the main sample. None of the outlooks has a statistically significant effect. Weaker effects before 2010 could indicate less skillful forecasts, less concern about the possibility of major events (later years have seen more billion dollar disasters (*22*)), less media attention, different market environments, or news leaking ahead of time before nondisclosure agreements became standard.

Supplementary figure 4 assesses the main results' robustness to the regression specification. The preferred specification (black) is the same as in Figure 2a of the main text. The preferred specification's controls help absorb the effects of any news not directly related to the seasonal outlooks without including controls (such as the S&P 500) that would absorb the effects of outlooks that move a wide array of firms. It also weights observations by the inverse of relative bid-ask spread in order to favor more liquid options. The robustness check (gray) reports a sparse specification that drops all controls other than the constant and dummies and does not weight observations. The point estimate is thus the simple average of the (suitably demeaned) outlook-day observations that are not within a day of earnings report releases. The two outlooks (NOAA June ENSO and NOAA Winter) that showed significant effects in the preferred specification have remarkably similar point estimates in the sparse specification. The point estimates are more sensitive to specification for the outlooks that did not show significant effects, although effects remain insignificant in the sparse specification.

Supplementary figure 5 assesses the main results' robustness to using options with the next-longest maturity. The results are rather consistent across the two expiration dates even though there are only around half as many usable firm-years with the longer expiration date. The June ENSO Outlook is again significant at the 5% level ($p = 0.014$). No other outlook approaches significance: in particular, the NOAA Winter Outlook's effect is no longer significant at a reasonable level, although its point estimate is still negative. One may be tempted to interpret the

term structure of the effects, but we caution against this reading. Seasonal outlooks might provide new information about future profits, but the options that expire closer to the forecasted seasonal climate need not respond to the outlooks any more strongly than options that expire much sooner. In either case, efficient markets should convert uncertainty about outlooks' information content into uncertainty about the present value of firms' dividends, and thus into uncertainty about stock prices at all times after the outlook's release and into higher-valued options at all expirations leading up to—and perhaps even after—the forecasted seasonal climate.

Supplementary figure 6 assesses the main results' robustness to using the nearest-to-the-money of the out-of-the-money put options, instead of using call options. The results are largely similar. The NOAA June ENSO Outlook is still significant at conventional levels ($p = 0.01$). As when varying options' maturities, no other outlook approaches significance at conventional levels: in particular, the NOAA Winter Outlook's point estimate is still negative but its p-value increases to 0.27.

Supplementary figure 7 assesses robustness to plausible ways of clustering standard errors. The main text clusters by date, on the argument that movements over time should be largely uncorrelated whereas movements by different firms on a given date could well be correlated. If we were concerned about correlation over time, we may instead cluster by firm-year. Supplementary figure 7 shows that standard errors shrink dramatically under this form of clustering (or when not clustering at all) and that two-way clustering by both date and firm-year yields results essentially identical to clustering by date alone. The effects of the June ENSO and Winter Outlooks are significant at the 1% level with the tighter standard errors produced by clustering by firm-year (or by not clustering), and it is still true that no other outlook approaches a significantly negative effect.

Figures 8 contains placebo tests. These plots repeat the analysis of Figure 2a from the main text, except pretending that outlooks were released anywhere from ten days before to ten days

after their true release dates. Across each panel's twenty fake dates, we should expect to see two dates be significantly negative at the 10% level merely by chance. This is approximately what we see. The June ENSO release date (labeled as day 0) has a smaller p-value than any of the 20 fake dates. The estimate on the Winter Outlook's true release date is less exceptional but still has a smaller p-value than all but 4 of the 20 fake dates (and it's worth noting that what we have classified as the day after the true date has a p-value of 0.05). This worse rank reflects both the larger p-value for the Winter Outlook's central estimate and the randomness of more of its fake dates happening to have unusually small p-values. In contrast, 15 of the 20 fake dates have a smaller p-value than does the NOAA Hurricane Outlook, reinforcing our failure to detect an effect of this outlook.

Supplementary figure 9 reports the value measures for each month's ENSO Outlook. For either measure, the June Outlook is significantly different from zero at the 1% level. For the risk reduction measure, only the May Outlook is also significant at the 10% level ($p = 0.093$), and for the option market premium, only the January Outlook is also significant at the 10% level ($p = 0.057$).

Finally, supplementary table 5 gives the numbers underlying Figure 3a in the main text.

Supplementary Discussion 6: Sensitivity of Value Calculations to Forecast Target

In the main text, we measure the ENSO Outlook's skill by the anomaly correlation coefficient for forecasts of an October-November-December (OND) target. Figures 10 and 11 assess sensitivity to a November-December-January (NDJ) target, a December-January-February (DJF) target, and a constant 5-month lead time target. The skill for each target is measured as the anomaly correlation coefficient from (*17*). The skill of each month's outlook varies across panels of a figure, but the estimated reductions in risk exposure and option market premia are

constant across panels. We see that skill jumps from the May to the June outlook regardless of the forecast target, whereas there is very little change in skill between the June and July outlooks regardless of forecast target.

Supplementary table 6 considers the implications for the value of a 1% improvement in ENSO prediction skill. The first column reports the anomaly correlation coefficients from (*17*). The June outlook's skill is similar across forecast targets, but the May outlook has greater skill at later forecast targets. If we based our value calculations on the later targets, then we would estimate greater value because a smaller jump in skill now explains the same jump in the market premium.

The remaining columns duplicate our value calculations with alternate forecast targets. The first row provides the values reported in the main text. The second and third rows show that measuring the May-to-June jump in skill from an NDJ or DJF target would increase our estimated value of the June ENSO outlook by 30–40%. The final row shows that using skill at a constant 5-month lead time (OND for the May outlook and NDJ for the June outlook) yields results that are similar to those reported in the main text. The values reported in the main text appear conservative.

Supplementary References

1. National Academies of Sciences, Engineering, and Medicine. *Next Generation Earth System Prediction: Strategies for Subseasonal to Seasonal Forecasts* (2016).

2. Kirtman, B. P. *et al.* The North American Multimodel Ensemble: Phase-1 Seasonal-to-Interannual Prediction; Phase-2 toward Developing Intraseasonal Prediction. *Bulletin of the American Meteorological Society* **95**, 585–601 (2014).

3. DelSole, T., Nattala, J. & Tippett, M. K. Skill improvement from increased ensemble size and model diversity. *Geophysical Research Letters* **41**, 7331–7342 (2014).

4. Lu, F. *et al.* GFDL's SPEAR seasonal prediction system: Initialization and ocean tendency adjustment (OTA) for coupled model predictions. *Journal of Advances in Modeling Earth Systems* **12**, e2020MS002149 (2020).

5. Barnston, A. G., Tippett, M. K., L'Heureux, M. L., Li, S. & DeWitt, D. G. Skill of real-time seasonal ENSO model predictions during 2002–11: Is our capability increasing? *Bulletin of the American Meteorological Society* **93**, 631–651 (2012).

6. Vecchi, G. A. *et al.* On the seasonal forecasting of regional tropical cyclone activity. *Journal of Climate* **27**, 7994–8016 (2014).

7. Murakami, H. *et al.* Simulation and prediction of category 4 and 5 hurricanes in the high-resolution GFDL HiFLOR coupled climate model. *Journal of Climate* **28**, 9058–9079 (2015).

8. Barnston, A. G., Tippett, M. K., Ranganathan, M. & L'Heureux, M. L. Deterministic skill of ENSO predictions from the North American Multimodel Ensemble. *Climate Dynamics* **53**, 7215–7234 (2019).

9. Kruttli, M., Roth Tran, B. & Watugala, S. W. Pricing Poseidon: Extreme weather uncertainty and firm return dynamics. Finance and Economics Discussion Series 2019-054, Board of Governors of the Federal Reserve System (2019).

10. Philander, S. G. H. El Niño Southern Oscillation phenomena. *Nature* **302**, 295–301 (1983).

11. Jia, L. *et al.* Improved seasonal prediction of temperature and precipitation over land in a high-resolution GFDL climate model. *Journal of Climate* **28**, 2044–2062 (2015).

12. Thomas, E. E., Vimont, D. J., Newman, M., Penland, C. & Martínez-Villalobos, C. The role of stochastic forcing in generating ENSO diversity. *Journal of Climate* **31**, 9125–9150 (2018).

13. L'Heureux, M. L. *et al.* ENSO Prediction. In McPhaden, M. J., Santoso, A. & Cai, W. (eds.) *El Niño Southern Oscillation in a Changing Climate*, 227–246 (Wiley, 2020).

14. Barnston, A. G. *et al.* Long-lead seasonal forecasts—Where do we stand? *Bulletin of the American Meteorological Society* **75**, 2097–2114 (1994).

15. McPhaden, M. J. Tropical Pacific Ocean heat content variations and ENSO persistence barriers. *Geophysical Research Letters* **30** (2003).

16. Yu, J.-Y. & Kao, H.-Y. Decadal changes of ENSO persistence barrier in SST and ocean heat content indices: 1958–2001. *Journal of Geophysical Research: Atmospheres* **112** (2007).

17. L'Heureux, M. L. *et al.* Strength outlooks for the El Niño-Southern Oscillation. *Weather and Forecasting* **34**, 165–175 (2019).

18. Almanac, F. How Does The Farmers' Almanac Predict The Weather? (2021). URL `https://www.farmersalmanac.com/predicting-weather`.

19. Walsh, J. E. & Allen, D. Testing the Farmer's Almanac. *Weatherwise* **34**, 212–215 (1981).

20. Gray, W. M. Atlantic seasonal hurricane frequency. Part I: El Niño and 30 mb quasi-biennial oscillation influences. *Mon. Wea. Rev.* **112**, 1649–1668 (1984).

21. Dubinsky, A., Johannes, M., Kaeck, A. & Seeger, N. J. Option pricing of earnings announcement risks. *The Review of Financial Studies* **32**, 646–687 (2019).

22. Smith, A. B. US billion-dollar weather and climate disasters, 1980–present. *NCEI Accession* **209268** (2020).

Supplementary Table 1: ENSO skill by month forecast is issued. Forecasts are from the North American Multi-Model Ensemble for an October-November-December target, so that a forecast issued in March (September) corresponds to a 7 (1) month lead time. Skill is measured as the anomaly correlation coefficient with observed (ERSSTv5) Nino3.4 index over 1982–2010. Table reproduced from Figure 2 in (*17*).

March	April	May	June	July	August	September
0.77	0.78	0.82	0.86	0.87	0.90	0.94

Supplementary Table 2: Release Dates of Seasonal Climate Outlooks, 2000–2019

	NOAA			Farmers' Almanac	Colorado State University
Year	June ENSO	Winter	Hurricanes	Winter	Hurricanes
2000		Thu Oct 12	Wed May 10		Fri Apr 7
2001	Mon Jun 11	Wed Oct 17	Mon May 21		Fri Apr 6
2002	Thu Jun 6	Thu Oct 17	Mon May 20		Fri Apr 5
2003	Thu Jun 12	Thu Oct 16	Mon May 19		Fri Apr 4
2004	Mon Jun 10	Thu Oct 21	Mon May 17		Fri Apr 2
2005	Thu Jun 9	Thu Oct 20	Mon May 16		Fri Apr 1
2006	Thu Jun 8	Thu Oct 19	Mon May 22		Tue Apr 4
2007	Thu Jun 7	Tue Oct 9	Tue May 22		Tue Apr 3
2008	Thu Jun 5	Thu Nov 20	Thu May 22		Wed Apr 9
2009	Thu Jun 4	Thu Oct 15	Thu May 21		Tue Apr 7
2010	Thu Jun 3	Thu Oct 21	Thu May 27	Mon Aug 30	Wed Apr 7
2011	Thu Jun 9	Thu Oct 20	Thu May 19	Mon Aug 29	Wed Apr 6
2012	Thu Jun 7	Thu Oct 18	Thu May 24	Mon Aug 27	Wed Apr 4
2013	Thu Jun 6	Thu Nov 21	Thu May 23	Mon Aug 26	Wed Apr 10
2014	Thu Jun 5	Thu Oct 16	Thu May 22	Mon Aug 25	Thu Apr 10
2015	Thu Jun 11	Thu Oct 15	Wed May 27	Mon Aug 17	Thu Apr 9
2016	Thu Jun 9	Thu Oct 20	Fri May 27	Mon Aug 15	Thu Apr 14
2017	Thu Jun 8	Thu Oct 19	Thu May 25	Mon Aug 14	Thu Apr 6
2018	Thu Jun 14	Thu Oct 18	Thu May 24	Mon Aug 27	Thu Apr 5
2019	Thu Jun 13	Thu Oct 17	Thu May 23	Mon Aug 26	Thu Apr 4

Supplementary Table 3: Economic News Released within One Week of NOAA Outlooks, 2000–2019

Year	June ENSO	Winter	Hurricanes
2000	N/A	+1(P)	-3(E),+2(P)
2001	+3(P)	+2(C)	-3(U1)
2002	+1(E)	+1(C)	-3(U1)
2003	+1(P)	**0(C)**	-3(C)
2004		-2(C),+1(U1)	-3(C)
2005		+1(U1)	+1(P),+2(C)
2006		-2(P),-1(C),+1(U1)	-2(U1)
2007		-2(E),+3(P)	
2008	+1(E)	-2(P),-1(C),+1(U1)	-2(P)
2009	-1(U2),+1(E)	**0(C)**	+1(U1)
2010	-1(U2),+1(E)	+1(U1)	
2011		-2(P),-1(C),+1(U1)	+1(U1)
2012		-2(C),+1(U1)	
2013	+1(E)	-1(C),**0(P)**,+1(U1)	
2014	+1(E)	-1(P)	
2015	+1(P)	-1(P),**0(C)**	**0(U1)**
2016		-2(C),+1(U1)	
2017		+1(U1)	
2018	-2(C),-1(F,P),+1(U1)	+1(U1)	
2019	-2(P),-1(C)	**0(U1)**	

Numbers in the table signify how many days before (-) or after (+) the NOAA outlook was the economic outlook released. Bolded values were released on the same day as the NOAA outlook. Cells are empty if none of the economic outlooks was released within 3 days on either side of the NOAA outlook.

C: Consumer Price Index, Real Earnings

E: Employment Situation Summary

F: Federal Open Market Committee Press Release

G: GDP Growth Estimate

P: Producer Price Index

U1: Regional and State Employment and Unemployment

U2: Metropolitan Area Employment and Unemployment

Supplementary Table 4: Results of estimating a common effect across all firms

	NOAA: June ENSO	NOAA: Winter Weather	NOAA: Hurricanes	Farmers' Almanac: Winter Weather	Colorado State University: Hurricanes
Estimate	-0.017	-0.0084	0.0028	-0.00029	0.0064
Standard Error	0.0075	0.0068	0.0069	0.0066	0.0069
p-value	0.013	0.11	0.66	0.48	0.82
Firms	2,955	3,145	2,639	2,957	3,062
Firm-Years	13,866	14,143	11,781	13,506	14,528
Observations	403,965	433,183	307,467	371,665	417,943

Supplementary Table 5: Market Capitalization (Millions of Dollars)

	All firms	All firms in sample	$p < .10$	$p < .05$	$p < .01$
June ENSO (NOAA)	43,749,960	33,278,935	13,362,705	9,212,309	4,088,519
Winter Weather (NOAA)	43,749,960	33,476,098	5,740,457	4,125,152	1,747,749
Hurricanes (NOAA)	43,749,960	32,502,331	4,061,097	2,759,047	1,155,423
Winter Weather (Farmers')	43,749,960	33,231,101	4,403,742	2,643,044	805,642
Hurricanes (CSU)	43,749,960	33,279,784	5,346,159	3,924,070	1,346,869

Excludes all equities with NAICS code 5259 ("Other Investment Pools and Funds"). Market capitalization is from 2019 when available and is from the most recent available year prior to 2019 otherwise.

Supplementary Table 6: Value of a 1% increase in ENSO skill.

	Outlook Skill		Value of 1% Skill Improvement	
	May	June	Risk Exposure ($billion)	Option Market Premium ($million)
OND Target	0.8193	0.8623	18.2	1.79
			(-16.4,52.8)	(-0.31,3.90)
NDJ Target	0.8342	0.8676	23.9	2.35
			(-21.5,69.3)	(-0.41,5.11)
DJF Target	0.8285	0.8591	25.9	2.55
			(-23.3,75.1)	(-0.44,5.54)
5-Month Target	0.8193	0.8676	16.2	1.60
			(-14.6,47.0)	(-0.28,3.47)

Skill measured as the anomaly correlation coefficient from (*17*).
95% confidence intervals in parentheses.

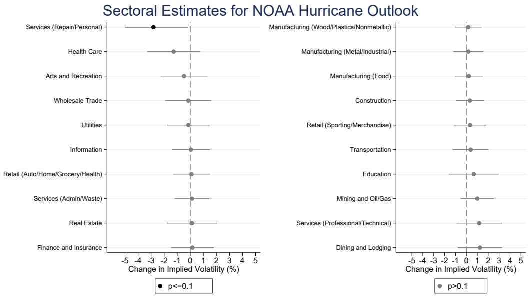

(a) NOAA Hurricane

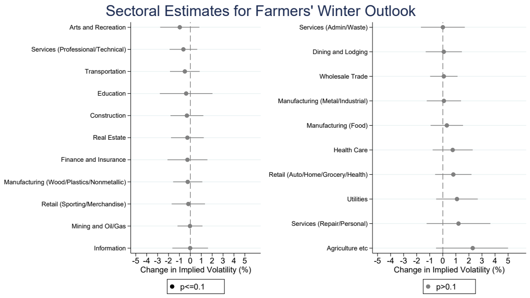

(b) Farmers' Winter

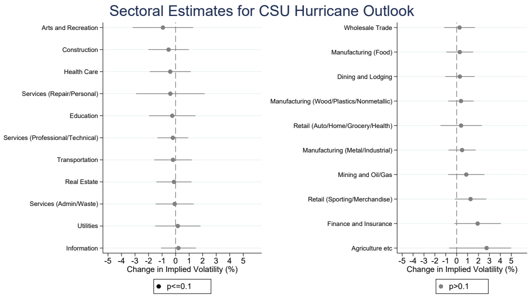

(c) CSU Hurricane

Supplementary Figure 1: Estimated effects of the (a) National Oceanic and Atmospheric Administration Hurricane, (b) Farmers' Almanac Winter, and (c) Colorado State University Hurricane Outlooks by sector, ordered by point estimates and with 95% confidence intervals. Black markers indicate that the estimate is significant at the 10% level.

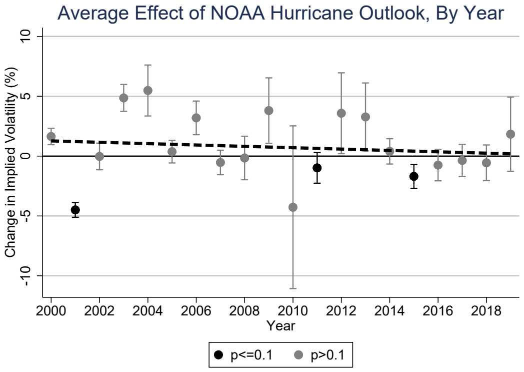

(a) NOAA Hurricane

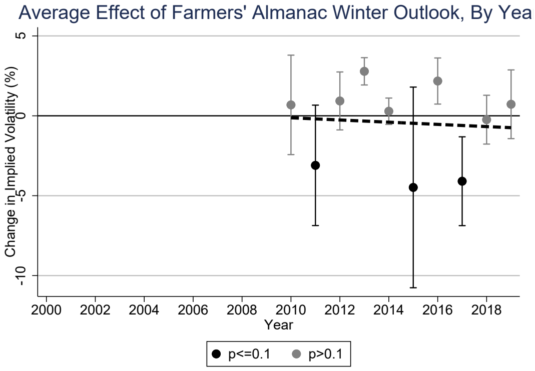

(b) Farmers' Winter

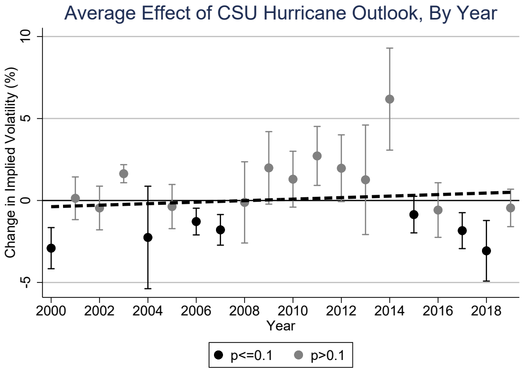

(c) CSU Hurricane

Supplementary Figure 2: Estimated effects of the (a) National Oceanic and Atmospheric Administration Hurricane, (b) Farmers' Almanac Winter, and (c) Colorado State University Outlooks by release year, with 95% confidence intervals. The dashed line is the trend line across all years' estimates. Black markers indicate that the estimate is significant at the 10% level.

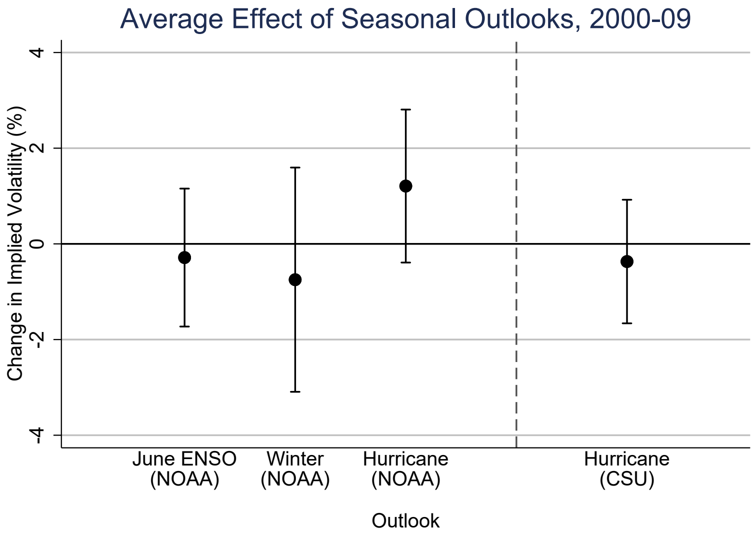

Supplementary Figure 3: Estimated average effects on implied volatility across all firms in 2000–2009, with 95% confidence intervals.

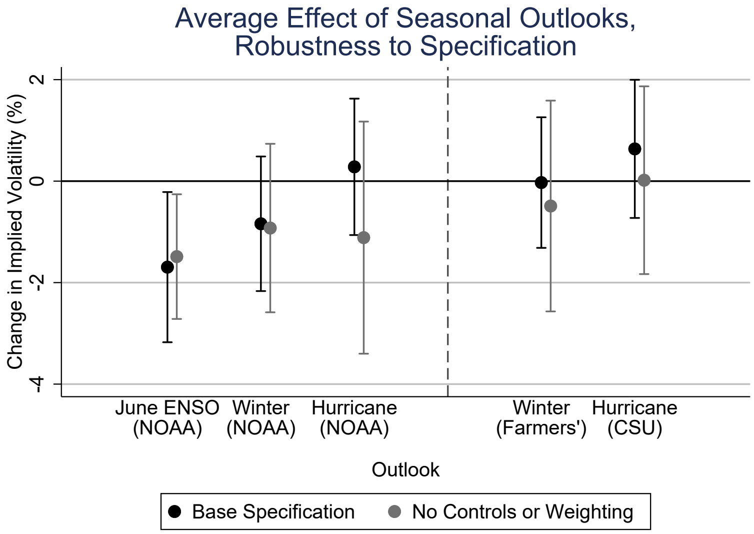

Supplementary Figure 4: Assessing robustness to regression specification.

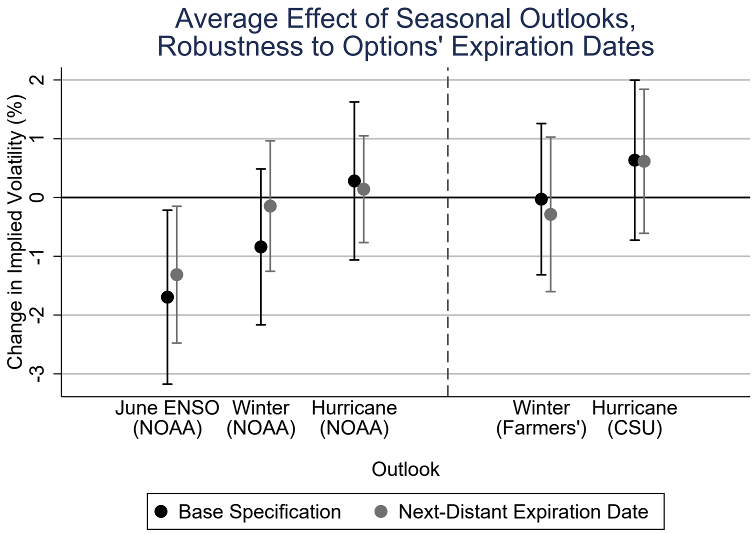

Supplementary Figure 5: Assessing robustness to expiration date.

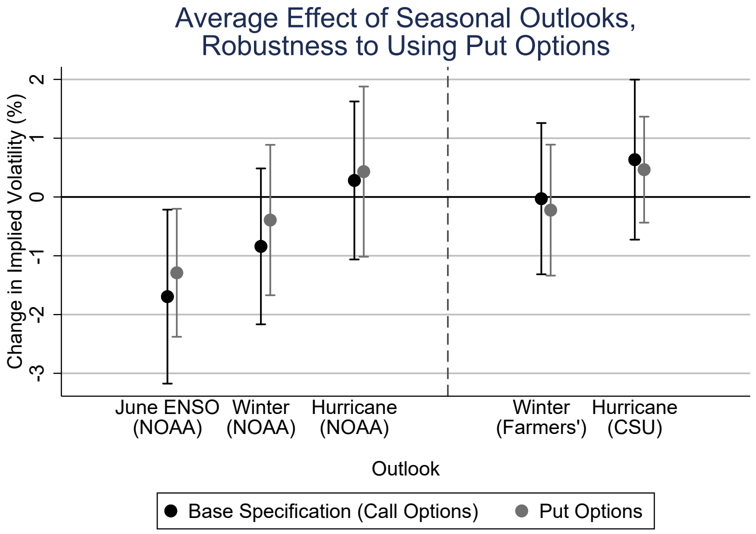

Supplementary Figure 6: Assessing robustness to using puts instead of calls.

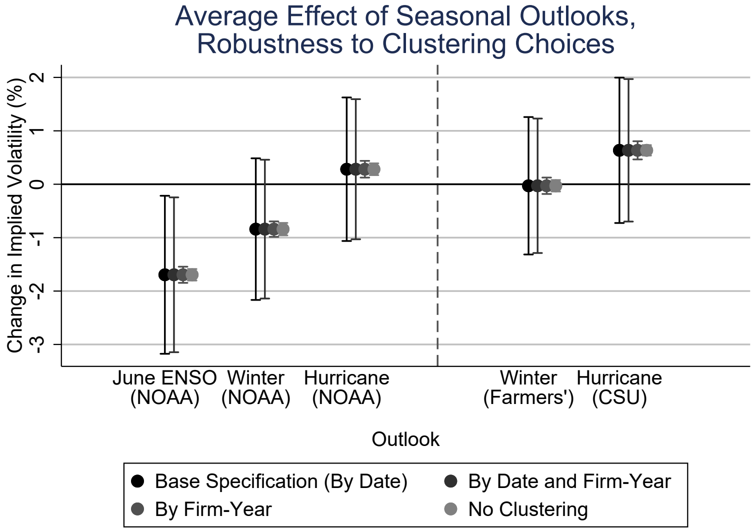

Supplementary Figure 7: Assessing robustness to level of clustering.

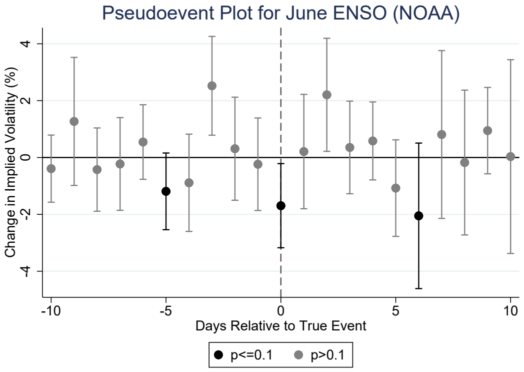

(a) Pseudoevent Plot for June ENSO Outlook

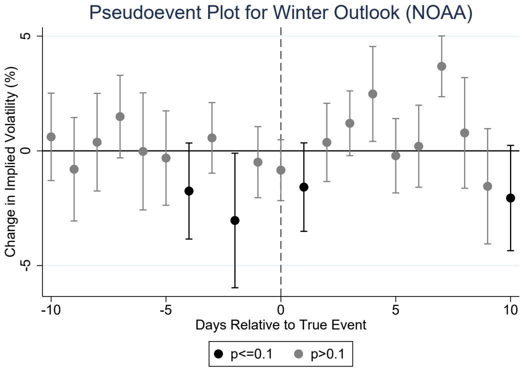

(b) Pseudoevent Plot for Winter Outlook

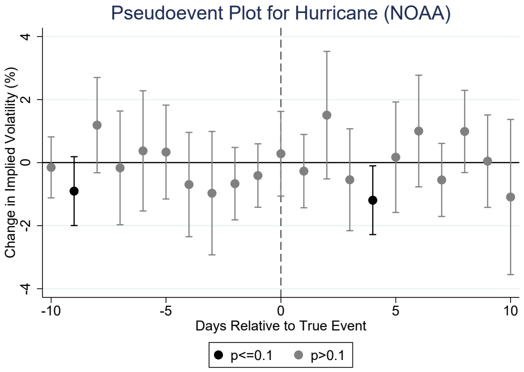

(c) Pseudoevent Plot for Hurricane Outlook

Supplementary Figure 8: Placebo tests, which pretend that the National Oceanic and Atmospheric Administration's (a) June El Niño Southern Oscillation, (b) Winter, and (c) Hurricane Outlooks were released earlier or later than they actually were. Black markers indicate that the estimate is significant at the 10% level.

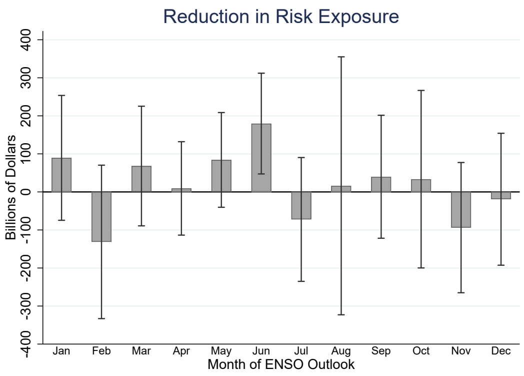

(a) Reduction in Risk Exposure

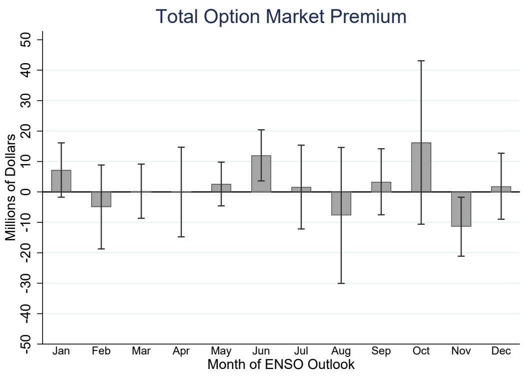

(b) Total Premium

Supplementary Figure 9: Measures of aggregate value for each month's June El Niño Southern Oscillation Outlook, with 95% confidence intervals. (a) Reduction in market capitalization exposed to a one standard deviation risk; (b) option market premium induced by an upcoming seasonal outlook.

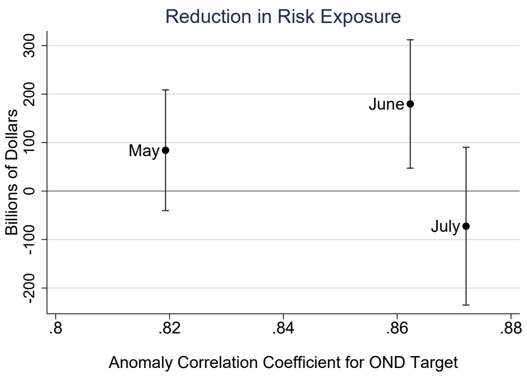

(a) OND Target (Base)

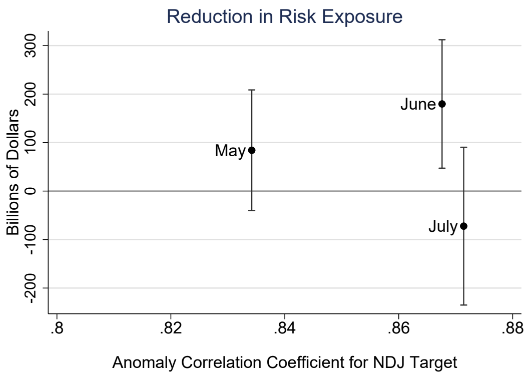

(b) NDJ Target

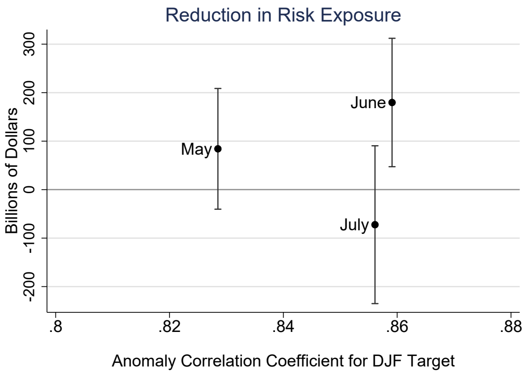

(c) DJF Target

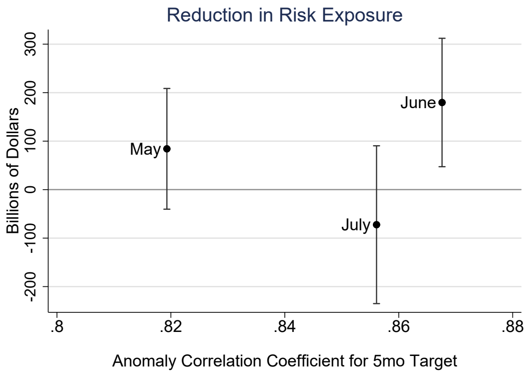

(d) 5-Month Target

Supplementary Figure 10: Reduction in risk exposure against skill at predicting different El Niño Southern Oscillation targets: (a) October-November-December, (b) November-December-January, (c) December-January-February, (d) 5 months ahead.

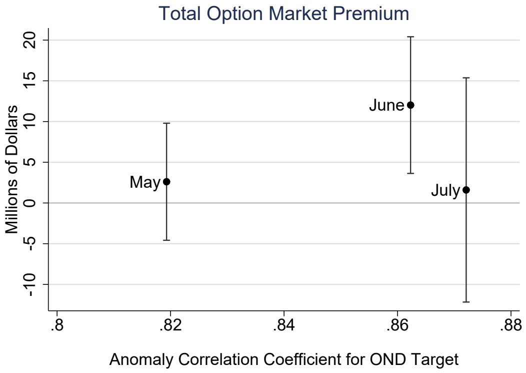

(a) OND Target (Base)

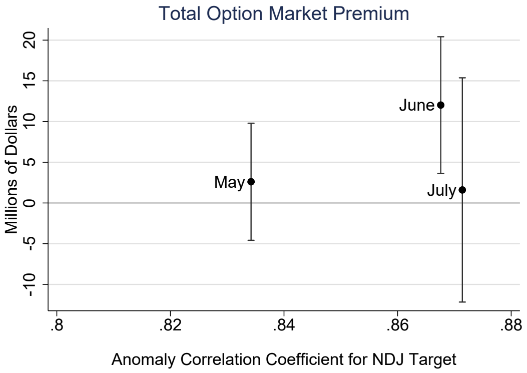

(b) NDJ Target

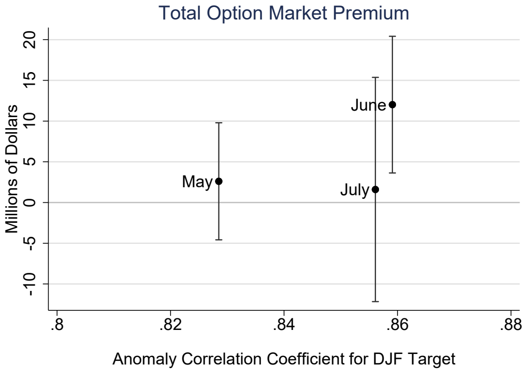

(c) DJF Target

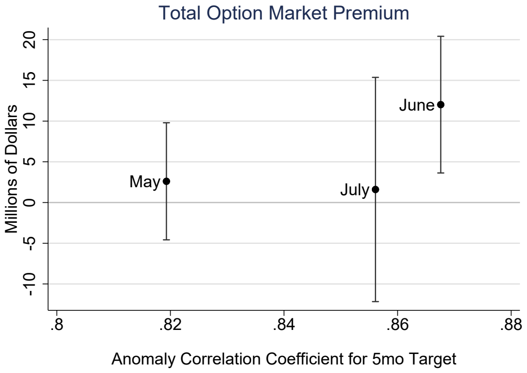

(d) 5-Month Target

Supplementary Figure 11: Option market premium against skill at predicting different El Niño Southern Oscillation targets: (a) October-November-December, (b) November-December-January, (c) December-January-February, (d) 5 months ahead.
